# Divergent mechanisms governing aboveground biomass in desert plants across a drought gradient

**DOI:** 10.1016/j.isci.2026.115304

**Published:** 2026-03-10

**Authors:** Kaiye Yuan, Hailiang Xu, Junjie Yan, Guangpeng Zhang

**Affiliations:** 1 State Key Laboratory of Desert and Oasis Ecology, Xinjiang Institute of Ecology and Geography, Chinese Academy of Sciences, Urumqi 830011, China; 2 University of Chinses Academy of Sciences, Beijing 100049, China; 3 College of Biology and Geography Science, Yili Normal University, Yining 835000, China

**Keywords:** natural sciences, plant biology, plant physiology, plant ecology, plant systematics, agricultural soil science

## Abstract

How plant traits and soil factors jointly regulate aboveground biomass (AGB) along soil moisture gradients is central to arid-land ecology. Along a soil moisture transect in China’s Kalamaili Nature Reserve, we integrated soil properties with trait-based community metrics to identify the drivers of AGB. AGB was regulated by soil water content (SWC) through both direct effects and indirect nitrogen-mediated pathways, whereby higher total nitrogen increased community-weighted maximum height. The AGB-SWC relationship was strongly nonlinear, with a clear threshold near 28% SWC: responses were weak below this point but increased sharply above it. Driver importance shifted with aridity, as the positive effect of plant height weakened under extreme drought, and its interaction with SWC shifted from synergistic in dry conditions to antagonistic in wetter conditions. These results reveal soil moisture thresholds and context-dependent trait-resource coupling that inform predictions and management of productivity in arid ecosystems.

## Highlights

• The aboveground biomass response to soil moisture is markedly nonlinear
• Soil moisture, nitrogen, and plant height drive aboveground biomass jointly
• Aridity shifts aboveground biomass drivers from water to functional trait
• Restoration priorities differ above/below the 28% moisture threshold

## Graphical abstract

**Figure:**
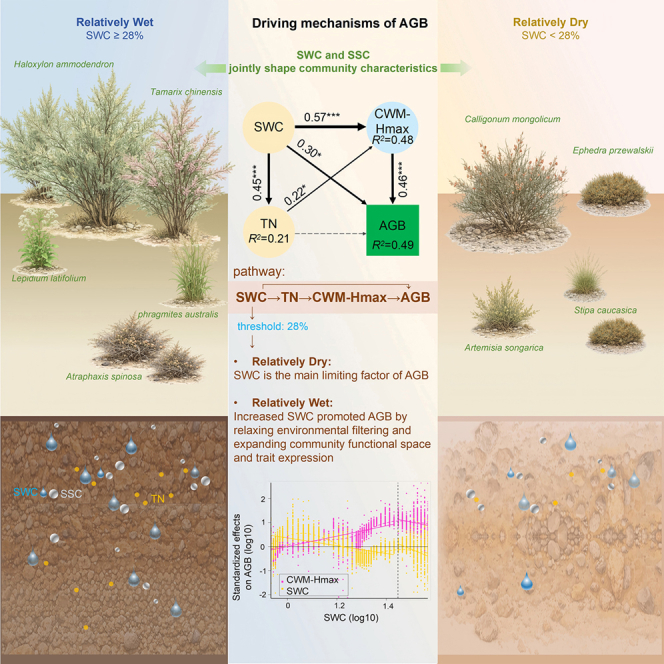

## Introduction

The acceleration of global aridification poses a critical challenge: Under persistent water scarcity, achieving precise prediction and enhancement of aboveground biomass (AGB) in drylands have become a central goal for ecosystem restoration and precision water management.^1^ As an integrative functional metric, AGB integrates the influence of soil properties and community structure and functioning, and is therefore a key indicator of carbon sink capacity, maintenance of vegetation cover, and ecosystem services in drylands.^2^^,^^3^^,^^4^^,^^5^ Observational data indicate that from 1960 to 2023, approximately 27.9% of the global land surface shifted significantly toward more arid states,^6^ with projections suggesting a further 7% increase in dryland area by 2100.^7^ Intensifying aridity precipitates abrupt structural and functional reorganizations at aridity index (AI) thresholds of approximately 0.54, 0.70, and 0.80.^8^^,^^9^ Collectively, these studies suggest that in drylands, both the key drivers of AGB and their underlying mechanisms are not static, but shift fundamentally across gradients of moisture stress.

The biodiversity-ecosystem functioning (BEF) relationship continues to elicit scientific discussion. Empirical evidence, including global syntheses,^10^ frequently reports a positive diversity-AGB relationship across ecosystems, explained by overyielding, covariance (complementarity/selection), and portfolio (variance-buffering) effects.^11^^,^^12^^,^^13^^,^^14^ Theoretically, this pattern is interpreted through two long-standing hypotheses. One is niche complementarity, where trait-based differences among species create positive biotic feedbacks—via resource partitioning, facilitation, or mutualisms—that improve resource-use efficiency and stability, ultimately enhancing AGB.^15^^,^^16^^,^^17^ The other is the selection effect (or mass-ratio hypothesis), which attributes ecosystem functioning largely to the dominance of highly productive species.^12^^,^^18^ Both perspectives can be quantified using plant functional traits: Functional diversity (FD) measures among-species trait differences,^19^ whereas community-weighted means (CWM) capture the trait values of dominant species.^20^ In arid ecosystems, AGB is tightly coupled with functional traits related to leaf economics, hydraulic architecture, roots, and mutualistic symbiosis.^21^^,^^22^^,^^23^^,^^24^ Near or beyond aridity thresholds, trait convergence strengthens, the mass-ratio contribution to AGB increases, and the influence of diversity weakens.^8^^,^^10^^,^^18^^,^^25^ Long-term studies in dryland forests and shrublands demonstrate that selection and niche complementarity effects co-occur, and their relative contributions to AGB shift with climate variability.^26^^,^^27^^,^^28^ Therefore, it remains unclear when, along aridity gradients, the dominant drivers of AGB undergo critical, threshold-like transitions, and despite accelerating global aridification, there is still no consensus on this issue.

In drylands, soil water content (SWC) is both the dominant stressor on plant communities and a pivotal factor that couples soil properties with plant community attributes.^29^^,^^30^^,^^31^ However, much of the research treats moisture as a static covariate,^32^^,^^33^^,^^34^ overlooking gradient-dependent, threshold-related nonlinear effects. At the same time, the selection of drivers is often limited to a single diversity metric,^18^^,^^35^ with insufficient identification of key structural causal pathways.^36^^,^^37^ Consequently, intrinsic regime shifts in driver mechanisms across gradients are obscured, leading to conflicting conclusions about what governs AGB.

To address these issues, we conducted a study along an arid-zone transect with heterogeneous soil environments and, using AGB as the focal response variable, systematically quantified the coupled relationships among soil properties, plant community attributes, and AGB. We focused on two questions: (1) at the soil and plant-community scales, what are the key drivers of AGB in desert vegetation? and (2) how do these drivers and their mechanisms shift along an aridity gradient, and does a moisture threshold exist? We propose two hypotheses: (1) based on resource limitation theory^38^—as soil drought intensifies, water limitation strengthens and becomes the dominant constraint on AGB accumulation; and (2) within the BEF framework’s niche complementarity theory^15^—as soil drought is alleviated, resource limitation relaxes and the expression range of plant functional traits expands, such that functional traits and diversity increasingly drive AGB accumulation. By integrating multiple modeling approaches with threshold diagnostics, this study provides empirical support for the coupled soil-plant-AGB cascade and identifies the moisture threshold marking critical shifts in the mechanisms driving AGB in dryland desert vegetation, thereby offering a clear theoretical basis and practical pathway for tailoring restoration strategies, enhancing carbon sequestration, and enabling precision water allocation in arid regions.

## Results

### Soil properties and plant community characteristics

We established 13 sampling sites along an SWC gradient spanning 38.8% ± 2.0% (S1) to 8.1% ± 0.1% (S13) (*n* = 65). One-way ANOVAs (Table 1) indicated significant among-site differences (*p* < 0.05) in soil physicochemical properties (SWC, SSC, TN, TP, and pH) and structural attributes (SP and PC), evidencing strong habitat heterogeneity across the study area.

**Table 1:** Mean soil physicochemical and structural properties at each sampling site (mean ± SD, *n* = 5 plots per site)

Site	SWC %	SSC mg/g	TN g/kg	TP g/kg	pH	SP %	PC %
S1	38.8 ± 2.0a	21.4 ± 2.3a	1.2 ± 0.3a	0.8 ± 0.1a	8.9 ± 0.1day	38.6 ± 0.2c	0.4 ± 0.0days
S2	26.6 ± 6.2b	0.8 ± 0.2c	0.7 ± 0.5b	0.7 ± 0.2 ab	9.0 ± 0.1day	42.4 ± 0.2a	0.8 ± 0.0a
S3	24.5 ± 2.3b	1.3 ± 0.2c	0.3 ± 0.1bc	0.7 ± 0.0b	9.5 ± 0.2 ab	42.2 ± 0.3a	0.8 ± 0.0 ab
S4	21.6 ± 1.6c	20.8 ± 2.6a	0.3 ± 0.1bc	0.5 ± 0.1de	8.4 ± 0.1ef	38.0 ± 0.1c	0.2 ± 0.0e
S5	18.8 ± 1.1day	0.4 ± 0.1c	0.3 ± 0.0c	0.5 ± 0.0cde	9.6 ± 0.2a	42.3 ± 0.3a	0.8 ± 0.0a
S6	18.6 ± 1.4days	11.1 ± 2.3b	0.5 ± 0.1b	0.7 ± 0.2bc	9.2 ± 0.2cd	41.6 ± 2.2 ab	0.7 ± 0.3b
S7	18.1 ± 0.1day	1.2 ± 0.2c	0.4 ± 0.1bc	0.7 ± 0.1b	9.2 ± 0.1cd	42.1 ± 0.2 ab	0.8 ± 0.0 ab
S8	10.9 ± 0.8e	2.0 ± 0.1c	0.2 ± 0.0c	0.5 ± 0.1e	9.0 ± 0.4days	41.9 ± 0.2 ab	0.7 ± 0.1 ab
S9	9.6 ± 1.9e	15.0 ± 2.1 ab	0.3 ± 0.0bc	0.7 ± 0.1b	9.3 ± 0.0bc	38.0 ± 0.2c	0.3 ± 0.1de
S10	9.3 ± 0.6e	5.8 ± 1.9bc	0.3 ± 0.0bc	0.4 ± 0.1e	8.2 ± 0.1f	41.8 ± 0.1 ab	0.6 ± 0.1c
S11	8.84 ± 0.2e	3.3 ± 0.9bc	0.7 ± 0.1b	0.7 ± 0.0 ab	8.6 ± 0.2e	41.8 ± 0.3 ab	0.7 ± 0.1abc
S12	8.8 ± 0.8e	1.3 ± 0.6c	0.3 ± 0.1bc	0.6 ± 0.1bcd	9.4 ± 0.3 ab	41.6 ± 0.3 ab	0.8 ± 0.0 ab
S13	8.1 ± 0.1f	1.1 ± 0.3c	0.4 ± 0.0bc	0.7 ± 0.0bc	9.2 ± 0.1cd	41.3 ± 0.2b	0.8 ± 0.0 ab

Different lowercase letters indicate significant differences among sites (one-way ANOVA followed by Tukey’s LSD test, *p* < 0.05). Sites sharing a common letter are not significantly different (*p* > 0.05). Data are presented as mean ± standard deviation (SD), with *n* = 5 plots per site. Lowercase letters indicate significant differences among sites for a given soil variable; means sharing a letter (a, b, or c) do not differ significantly (*p* > 0.05), and letters are ordered from highest to lowest (a > b > c).

**Figure 1:**
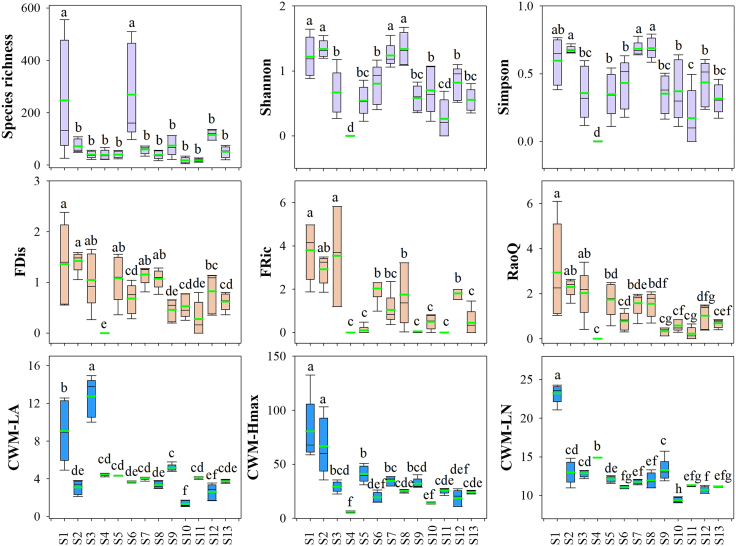
Comparisons of species diversity, functional diversity, and community-weighted means among sampling sites The figure shows the median, lower quartile (25%), and upper quartile (75%) for each of the data. Whiskers represent minimum and maximum values that are 1.5×IQR from the lower/upper quartile. The green line inside each box represents the mean value. Lowercase letters in the figure indicate the significance of differences in characteristics between community types, from largest to smallest, expressed as a > b. There was no significant difference (*p* > 0.05, Tukey’s LSD test) between the two community types that shared a common letter (a, b).

Along the SWC gradient, we recorded 23 vascular plant species from 10 families and 23 genera (Table S1). Species diversity, FD, CWM, and AGB differed significantly among sites (Figure 1). At S1—the site with the highest SWC—the Haloxylon-Phragmites-Tamarix community showed significantly higher values than other sites for the species richness index (except S6), FDis (except S2), FRic (except S3), Rao’s Q (except S2), CWM-LA (except S3), CWM-Hmax (except S2), and CWM-LN (except S4). The single-species Stipa glareosa community at S4 had the lowest species and FD and exhibited a dwarf growth form (lowest CWM-Hmax).

**Figure 2:**
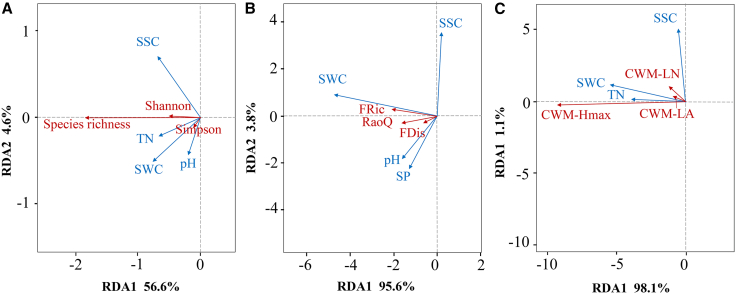
RDA ordinations linking plant community attributes to soil factors (A) RDA ordination of taxonomic diversity metrics in relation to soil factors across sampling sites. (B) RDA ordination of functional diversity metrics in relation to soil factors across sampling sites. (C) RDA ordination of community-weighted mean traits in relation to soil factors across sampling sites. Blue arrows indicate soil variables with significant correlations (*p* < 0.05). Red arrows denote plant variables.

We used ordination analyses to quantify the effects of soil factors on plant community attributes (Figure 2). SWC (*p* < 0.01), SSC (*p* < 0.05), TN (*p* < 0.05), and pH (*p* < 0.05) emerged as key soil determinants of species diversity (first two axes explained 39.3% of the variance; F = 5.3, *p* = 0.004). Species richness increased significantly with SWC, whereas Shannon and Simpson indices showed weaker responses. SWC (*p* < 0.01), SSC (*p* < 0.05), SP (*p* < 0.05), and pH (*p* < 0.1) were the principal soil drivers of FD (first two axes explained 44.9% of the variance; F = 6.7, *p* = 0.002). FD (FRic, RaoQ, FDis) was positively associated with SWC and negatively associated with SSC. SWC (*p* < 0.01), SSC (*p* < 0.1), and TN (*p* < 0.05) were the key soil predictors of CWM (first two axes explained 53.6% of the variance; F = 9.5, *p* = 0.002). CWM-Hmax was positively related to SWC and negatively related to SSC, whereas CWM-LA and CWM-LN responded more weakly. Taken together, SWC and SSC made the largest—and opposing—contributions to plant community attributes.

### Attribution of aboveground biomass to drivers

Building on the above analyses, plant community attributes were closely associated with soil factors across the study area. To elucidate how soil and plant variables regulate AGB accumulation, we used structural equation modeling (SEM) for path analysis. Prior to model fitting, we (1) used correlation analyses to screen variables significantly associated with AGB, (2) evaluated their predictive importance with random forest models, and (3) assessed multicollinearity to ensure a well-specified SEM.

**Figure 3:**
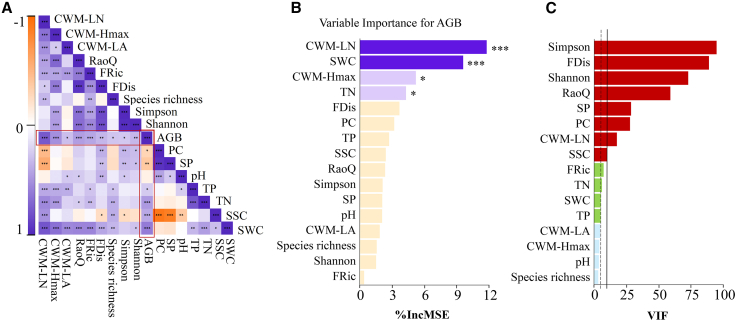
Key factors influencing AGB A is a heatmap of Pearson’s correlation coefficients between soil and plant factors. Each square represents the correlation between the two corresponding indicators on the horizontal and vertical axes. The color depth indicates the magnitude of Pearson’s correlation coefficient, with purple representing a positive correlation and orange representing a negative correlation. Values closer to ±1 indicate a stronger correlation, while values closer to 0 indicate a weaker correlation. B is the importance of each factor in predicting AGB. C is a collinearity analysis of factors. Solid black lines indicate factors with VIF >10, indicating severe multicollinearity issues. Dotted black lines indicate factors with VIF >5, indicating moderate multicollinearity issues. Significance is only displayed when *p* < 0.05. An asterisk indicates the level of significance. ∗*p* < 0.05, ∗∗*p* < 0.01, and ∗∗∗*p* < 0.001.

Figure 3A presents a Pearson’s correlation heatmap for relationships between soil factors, plant factors, and AGB. With the exception of pH, all selected soil and plant variables were significantly associated with AGB (*p* < 0.05). Notably, soil structural variables (SP and PC) were negatively correlated with AGB (*p* < 0.05), whereas the remaining variables were positively correlated. The random forest model identified CWM-LN, SWC, CWM-Hmax, and TN as key predictors with significant effects on AGB (*p* < 0.05; Figure 3B). In addition, strong correlations were evident among plant variables: Within species diversity, the Simpson and Shannon indices were highly positively correlated (*p* < 0.001), but neither was significantly correlated with species richness. Among FD metrics, FDis, FRic, and RaoQ were all strongly and positively intercorrelated (*p* < 0.001); and among CWM, CWM-LA, CWM-Hmax, and CWM-LN were significantly positively correlated with one another (*p* < 0.05). Considering species diversity versus trait attributes, Simpson and Shannon showed concordant trends and were strongly positively correlated with all FD metrics (FDis, FRic, and RaoQ) and with CWM-Hmax (*p* < 0.001); by contrast, species richness was positively correlated only with FRic and CWM-LN (*p* < 0.01). FRic was strongly and positively associated with all CWM metrics (CWM-LA, CWM-Hmax, and CWM-LN) (*p* < 0.001), whereas FDis and RaoQ showed similar patterns: Both were significantly positively correlated with CWM-Hmax and CWM-LN (*p* < 0.01) but not significantly related to CWM-LA. The high intercorrelations among variables indicated multicollinearity among key predictors of AGB. After excluding variables with VIF >5 based on variance inflation factor diagnostics (Figure 3C), SWC, CWM-Hmax, and TN were retained for SEM construction.

**Figure 4:**
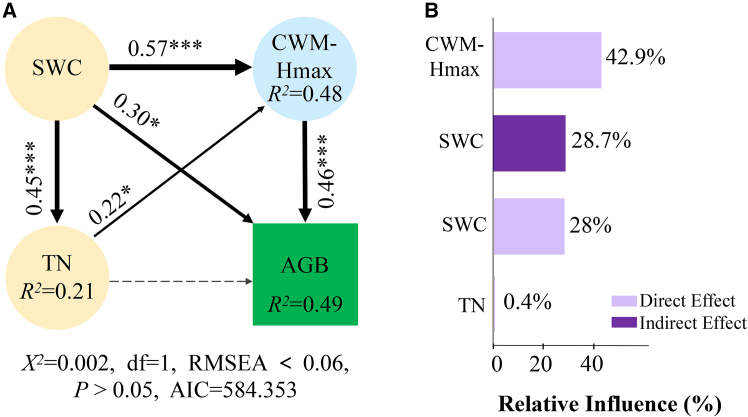
Structural equation model (SEM) examining direct and indirect relationships among SWC, TN, CWM-Hmax, and AGB A is a path diagram of the SEM. Solid and dashed lines indicate significant and non-significant relationships, respectively. Black lines denote positive correlations. Numbers beside arrows represent standardized path coefficients, with arrow thickness and numerical values reflecting relationship strength. χ^2^, chi-square statistic; df, degrees of freedom; RMSEA, root-mean-square error of approximation; *p*, *p*-value; AIC, Akaike information criterion. Asterisks denote the significance level of each coefficient: ∗ indicates significance at the 0.05 confidence level; ∗∗∗ indicates significance at the 0.001 confidence level. Bar charts represent the relative influence of each factor on AGB (B).

The SEM provided an excellent fit to the data (Figure 4A; χ^2^ = 0, CFI >0.95, RMSEA <0.06) and indicated that SWC, TN, and CWM-Hmax together explained 49% of the variation in AGB. Specifically, CWM-Hmax was the primary driver of AGB (path coefficient = 0.46, *p* < 0.001), with a relative contribution of 42.9% (Figure 4B). SWC ranked second: It directly promoted AGB accumulation (standardized path coefficient β = 0.30, *p* < 0.05) and also exerted a significant positive indirect effect on AGB via CWM-Hmax (total standardized indirect effect = 0.31, *p* < 0.01). The relative contributions of the direct and indirect effects were 28.0% and 28.7%, respectively. In addition, TN significantly increased CWM-Hmax (path coefficient = 0.22, *p* < 0.05) and thus affected AGB indirectly; its direct effect on AGB was not significant (relative contribution 0.4%). Taken together, SWC emerges as the key factor improving soil habitat conditions and community functional attributes, thereby promoting AGB accumulation. Acting as an intermediate variable, TN enhances community functional attributes, whereas CWM-Hmax serves as the critical bridge linking soil properties to community biomass and is the most proximate determinant of AGB.

### Soil moisture threshold for aboveground biomass

**Figure 5:**
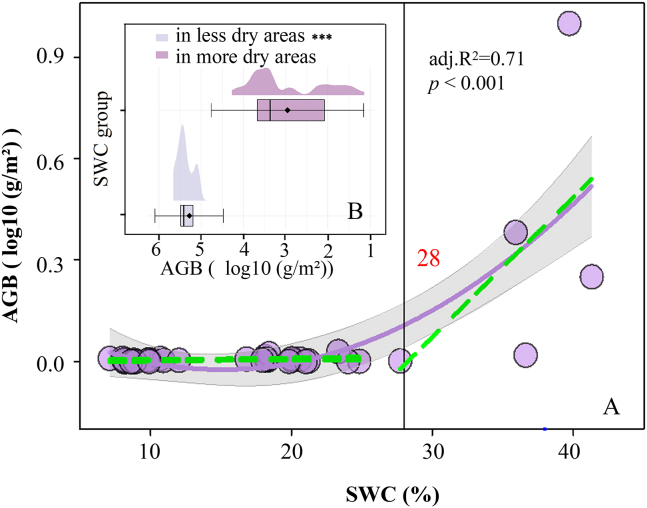
Responses of AGB to SWC A illustrates the response of AGB to SWC. The solid purple curve represents the nonlinear trend fitted using the generalized additive model (GAM). The black solid line and red inset icon indicate the identified SWC threshold. The green dashed lines represent linear fits on both sides of the threshold. B divides quadrats with SWC <28% into relatively arid zones (*n* = 52) and those with SWC ≥28% into relatively humid zones (*n* = 13), using generalized linear models for likelihood ratio tests to determine the significance of AGB differences between relatively arid and humid zones. Results are visualized using a raincloud plot. The significance level is ∗∗∗*p* < 0.001.

Given that SWC is the dominant factor structuring soil habitat and plant community attributes, we further examined how the SWC gradient modulates the mechanisms driving AGB. We first fitted a linear model and generalized additive models (GAMs) to characterize the overall response of AGB to SWC. Based on AIC (linear model: 193.48; GAM: 153.81), the GAM provided the best fit for the AGB-SWC relationship (Figure 5A; deviance explained = 73.81%, adj. R^2^ = 0.71, *p* < 0.001), revealing a pronounced nonlinear increase in AGB with rising SWC. Piecewise regression indicated a significant shift in the AGB-moisture response at SWC = 28%, which can be regarded as a critical moisture threshold linked to community degradation risk in the study area. A likelihood-ratio test (LRT) and the Davies test confirmed a significant difference in regression slopes across the threshold (χ2 = 13.33, df = 1, *p* < 0.001), validating the breakpoint (Figure 5B). Accordingly, plots were partitioned into two groups by SWC: a relatively arid set (SWC <28%, *n* = 52) and a relatively mesic set (SWC ≥28%, *n* = 13). In the relatively arid group, AGB responded weakly to increases in SWC (slope = 0.03 ± 0.01), whereas in the relatively mesic group the response intensified markedly (slope = 5.55 ± 0.36). Thus, once the moisture threshold is exceeded, the positive effect of water availability on AGB accumulation increases substantially.

**Figure 6:**
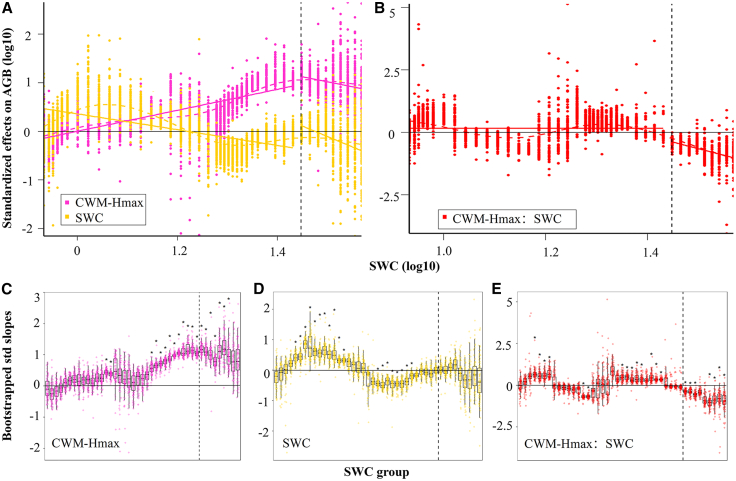
Nonlinear changes in the relationships between CWM-Hmax, SWC, their interaction, and AGB along aridity gradients Nonlinear changes in the standardized coefficients of CWM-Hmax and SWC (A) and their interaction (B) obtained from a linear model (Equation 11) across a moving subset window of field sampling sites surveyed along aridity gradients. The dots indicate the bootstrapped coefficients of the fixed terms for each subset window. The dashed lines represent the nonlinear trends fitted by generalized additive models (GAMs). The vertical dashed lines indicate the identified SWC thresholds, and the solid lines represent the linear fits on both sides of each threshold. C, D, and E Boxplots demonstrate the distribution of bootstrapped standardized coefficients corresponding to those in (A, B) for each subset window (*N* = 500 independent simulations). The boxplots show the median (center line), 25th, and 75th percentiles of each distribution. The whiskers represent the minimum and maximum values within 1.5 times the interquartile range below or above the median. Asterisks indicate coefficients significant at 95% confidence intervals (one-sided *p* ≤ 0.05).

Next, we applied a moving-window analysis to quantify the local marginal effects of CWM-Hmax, SWC, and their interaction on AGB across successive subsets along the soil-dryness gradient. Because TN lies downstream of SWC in the causal pathway, we excluded TN from the model to avoid over-controlling a mediator and biasing the SWC effect. The model explained 64.91% of the variance in AGB (Table 2); in 83% of windows, the 95% CI of the standardized slope did not cross zero. The results show that the soil-dryness gradient significantly modulates the relationships of SWC, CWM-Hmax, and their interaction with AGB (Figure 6). As SWC increased, the positive association between CWM-Hmax and AGB strengthened, but it weakened beyond the threshold (SWC = 28%) (Figure 6A). Window-wise tests of the standardized slopes indicated that the positive CWM-Hmax-AGB relationship was significant near the threshold (Figure 6C). With alleviating soil dryness, the SWC-AGB relationship exhibited a complex nonlinear pattern (Figures 6A and 6D). For SWC <18%, the relationship was predominantly and significantly positive; between 18% and 23% SWC, it became predominantly and significantly negative; between 23% and 36% SWC, it turned positive again and strengthened. The SWC × CWM-Hmax interaction related to AGB was predominantly and significantly positive in the relatively arid range ((Figures 6B and 6E), but predominantly and significantly negative in the relatively mesic range. In other words, as soil dryness intensifies, AGB becomes primarily governed by SWC, whereas the standalone effect of CWM-Hmax on AGB weakens.

**Table 2:** Linear models for the relationships between AGB, SWC, and CWM-Hmax

Variable	Estimate	Std. Error	t value	*p* value	VIF
Intercept	−6.75	2.31	−2.88	0.005	
CWM-Hmax	5.57	1.69	3.28	0.0015	2.2
SWC	6.21	1.52	4.08	0.0001	4.1
CWM-Hmax:SWC	−3.15	1.06	−2.96	0.004	4.9
Adjusted R^2^:0.637			F-statistic:42.02 (3, 68 df)

## Discussion

### Driving mechanisms of AGB in arid zone plant communities

Our results show that in this extremely arid ecosystem, SWC is the primary driver of variation in AGB, and the CWM-Hmax is the key trait through which plants respond to SWC and thereby modulate AGB. SWC exerts a significant direct effect on AGB and a significant indirect effect by increasing TN and thereby enhancing the expression of CWM-Hmax. This finding corroborates the hierarchical pathway SWC → TN → CWM-Hmax → AGB, indicating that soil moisture plays a pivotal role in regulating AGB in dryland plant communities. Accordingly, hypothesis 1 received strong support in the relatively arid portion of the study area. The SWC-AGB relationship is positively asymmetric, with sensitivity declining as aridity intensifies (Figure 5). This is consistent with the stress-gradient hypothesis, which posits that facilitative effects of abiotic factors become relatively more important under harsher conditions.^39^ In relatively mesic areas, increases in soil moisture elevate resource supply (e.g., nutrients and microbial activity), leading to greater-than-expected AGB accumulation.^40^^,^^41^ In relatively arid areas, drought-specialist species are more prevalent (e.g., higher water-use efficiency, thicker leaves, and down-regulated maximum photosynthetic capacity); such species minimize costs to tolerate water scarcity, resulting in lower biomass.^29^^,^^42^^,^^43^

Considering soil properties (Figure 3A), SWC is the key driver of habitat heterogeneity; its significant positive correlations with SSC and TP indicate that moisture fluctuations co-vary with salt accumulation and nutrient supply. At the same time, variation in SSC markedly influences soil chemistry and structure, evidenced by significant negative correlations with pH, total porosity, and connectivity, which suggests that salinity constrains the rhizosphere microenvironment and resource diffusion via coupled chemical-structural effects. Moreover, soil chemistry exhibits clear nutrient co-variation: pH correlates positively with TP, correlating strongly and positively with TN. Interestingly, although SWC and SSC may co-vary at the landscape scale, the RDA identified them as two opposing constraints on community attributes: FD (FRic, RaoQ, FDis) and CWM-Hmax increased with SWC but declined with SSC (Figure 2). This water-salinity antagonism further clarifies the AGB driver mechanism: Community functional space and trait composition emerged as outcomes of environmental filtering by SWC. increasing SWC relaxed this filtering, allowing functional space and trait expression to expand and thereby promoting AGB accumulation, whereas SSC suppresses trait expression and resource assimilation via chemical-structural constraints.

Together, these forces determine the positively asymmetric AGB response observed along the SWC gradient. In sum, the direct effect of SWC on AGB, together with its indirect effects mediated by soil habitat and plant traits, constitutes the main pathway of AGB variation; in relatively arid areas, trait convergence in drought-tolerant plants and salinity constraints limit AGB accumulation, explaining the reduced sensitivity of AGB to SWC. A meta-analysis of 28 global experiments found that aboveground net primary productivity (ANPP) is more sensitive to precipitation increases than to decreases.^44^ Knapp et al.^41^ similarly reported much stronger ANPP responses in wet years than in drought years. Our findings further corroborate the positive relationship between soil moisture and plant productivity. Moreover, we extend this positively asymmetric relationship to naturally assembled desert plant communities.

Among plant drivers, SR, FD, and CWM each showed significant positive associations with AGB (Figure 3A). Notably, the random forest importance analysis, collinearity diagnostics, and SEM results identified Against this background of SWC-driven environmental filtering, CWM-LN and CWM-Hmax as the strongest predictors of AGB (Figures 3 and 4). The correlations of SR and FD with AGB largely stemmed from their being driven by SWC and their co-variation with dominant plant traits, rather than from independent causal effects on AGB. Therefore, we prefer to regard SR and FD as “covarying indicators” reflecting soil moisture and nutrient conditions, rather than as dominant factors directly controlling AGB. This understanding aligns with the mass ratio hypothesis in the context of strong environmental filtering in arid regions, where ecosystem functioning is primarily determined by the trait characteristics of a few dominant species.^18^^,^^45^^,^^46^^,^^47^ CWM-LN represents the community means leaf nitrogen concentration, typically linked to maximal photosynthetic rates, protein synthesis, and metabolic activity. Higher LN confers greater carbon assimilation per unit leaf area and thus facilitates biomass formation.^48^^,^^49^ CWM-Hmax captures the community mean plant height, directly affecting light capture and canopy stratification.^50^^,^^51^ In drylands, taller plants often pair with deeper or more effective root systems, promoting reliable access to water and nutrients.^52^^,^^53^ Consequently, increases in the community means of these traits, this “mass ratio effect” more readily translates into gains in AGB.^54^^,^^55^^,^^56^^,^^57^

### Shift in AGB driving mechanisms along the drought gradient

Based on threshold diagnostics of the nonlinear SWC-AGB relationship, we identified 28% SWC as the breakpoint at which AGB shifts abruptly. Using a moving-window approach along the SWC gradient to estimate local marginal effects, we found that the influences of CWM-Hmax, SWC, and their interaction on AGB change markedly with moisture availability. As drought stress intensifies, the positive association between CWM-Hmax and AGB weakens. The local slope of SWC on AGB is nonlinear—significantly positive when SWC <18%, significantly negative at 18–23% SWC, and positive again and strengthening at 23–36% SWC—whereas the CWM-Hmax × SWC interaction is positive in the relatively arid range and negative in the relatively mesic range. These patterns indicate that the facilitative effect of CWM-Hmax on AGB accumulation is contingent on soil moisture and shifts across the aridity gradient.

In the arid zone, water limitation reduces diversity and weakens interspecific competition, thereby allowing taller species—with their superior water-use capacity and resource access—to directly translate trait advantages into increased AGB, resulting in a positive CWM-Hmax × SWC interaction. Consistent with hypothesis 2 in the mesic range, increased soil N and P permit a greater number of species to meet their requirements and fill niche space,^58^ which in turn intensifies competition for resources, including soil water, nutrients, and light. Beyond the moisture threshold, further increases in SWC introduce additional risks,^24^^,^^59^ including higher individual maintenance costs (support and hydraulic transport) and elevated cavitation risk (taller stems are more prone to embolism), which weaken the marginal contribution of CWM-Hmax to AGB and shift its interaction with SWC from synergistic to antagonistic. These patterns accord with the stress-gradient hypothesis—that facilitation is more prevalent under harsh conditions, whereas competition and structural costs rise as stress abates,^60^^,^^61^ and here we quantify this explicitly at the trait × environment level: The interaction is positive in the relatively arid zone but turns negative in the relatively mesic zone.

The local effect of SWC on AGB transitioned from negative (18–23% SWC) to positive (23–36% SWC), and back to negative beyond 36% SWC. RDA and correlation analyses of community attributes versus soil factors suggest that increases in soil moisture are accompanied by salt redistribution and reduced aeration, which may suppress nutrient transport and microbial activity in the rhizosphere,^62^ thereby constraining the gains in AGB attributable to community diversity and trait expression. As SWC rises further toward the threshold, it appears to promote AGB accumulation by substantially increasing CWM-Hmax and CWM-LN, although this step warrants additional confirmation in subsequent analyses. Furthermore, we observed declines in both SR and FD in relatively arid areas, indicating a contraction in the species pool and functional space under high environmental stress. In this context, community functioning was maintained primarily by a few species possessing key traits, as the community became disproportionately reliant on them. This shift is consistent with findings from similar latitudes, where intensifying stress weakens the positive effects of diversity (SR, FD) on stability while strengthening the mass-ratio effect.^63^^,^^64^ Notably, both the random forest and SEM converge on CWM, particularly CWM-Hmax and CWM-LN, as the key drivers of AGB, whereas the positive influence of diversity is expressed chiefly indirectly by elevating community means of key traits, with its strength highly sensitive to the moisture regime.

Building on these empirical patterns, we propose restoration guidelines for desert ecosystems aligned with the soil-dryness gradient: Maintaining SWC ≈28% should facilitate the addition of species that broaden functional space and elevate community means of key traits, yielding gains in AGB. Within the 18–23% SWC transition zone, simultaneously controlling salinity and enhancing pore connectivity can prevent losses in the contributions of community diversity and trait expression to AGB. By synthesizing the pathway from resource limitation to trait mediation and ultimately to AGB within a unified framework, our work elucidates the key drivers of AGB and their context-dependent dynamics. This integrated understanding provides a scientific foundation for precision water management and targeted vegetation restoration in extremely arid ecosystems.

### Limitations of the study

This study has several limitations that should be interpreted with caution. Our dataset was collected along a landscape-scale spatial gradient; although the integration of random forest importance, collinearity diagnostics, RDA, and SEM provides statistical support for the pathway “SWC → TN → CWM-Hmax → AGB,” this inferred causal chain may still be influenced by unmeasured covariates (e.g., microtopography, legacy disturbances, and groundwater depth) that co-vary with soil moisture. The identified SWC thresholds (28% and 18–23%) may vary with precipitation, evaporative demand, soil texture, and salt redistribution. Thus, assessing their spatiotemporal robustness will require multi-season and multi-year monitoring, coupled with validation at independent sites. In addition, while our study area represents a typical desert ecosystem, extrapolation to other drylands with different dominant species assemblages, soil types, and salinization mechanisms should be made cautiously. Future work that combines controlled water and salinity manipulations, continuous monitoring of soil moisture and aeration, and cross-region comparisons will be essential to better constrain the generality of these thresholds and to strengthen the scientific basis for management recommendations.

## Resource availability

### Lead contact

Further information and requests for resources should be directed to and will be fulfilled by the lead contact, Guangpeng Zhang (zgp@ms.xjb.ac.cn).

### Materials availability

This study did not generate new unique reagents.

### Data and code availability

The original data generated during the lab-based study of collected soil properties and vegetation survey are available at Mendeley Data: https://doi.org/10.17632/fyjshsnzyt.1.

This study does not report original code.

Any additional information required to reanalyze the data reported in this paper is available from the lead contact upon request.

## Acknowledgments

The authors thank the members of the laboratory for their insightful discussions on the drivers of aboveground biomass in desert ecosystems. Appreciation is also extended to collaborators and funding bodies for their support in advancing this research. The work was funded by the 10.13039/100009110Natural Science Foundation of Xinjiang Uygur Autonomous Region (No. 2022D01B214).

## Author contributions

The authors undertook different tasks for this paper. K.Y. performed data processing and wrote the manuscript. K.Y., H.X., J.Y., and G.Z. analyzed the data and provided direction for the research. All authors have read and agreed to the published version of the manuscript.

## Declaration of interests

The authors declare no conflict of interest.

## STAR★Methods

### Key resources table

**Table:** 

REAGENT or RESOURCE	SOURCE	IDENTIFIER
**Deposited data**		

Raw sequence data	This paper	https://doi.org/10.17632/fyjshsnzyt.1

**Software and algorithms**		

R 4.0.3	R Core Team	https://www.r-project.org
vegan	CRAN	https://cran.r-project.org/package=vegan
FD	CRAN	https://cran.r-project.org/package=FD
randomForest	CRAN	https://cran.r-project.org/package=randomForest
lavaan	CRAN	https://cran.r-project.org/package=lavaan
semPlot	CRAN	https://cran.r-project.org/package=semPlot
mgcv	CRAN	https://cran.r-project.org/package=mgcv
segmented	CRAN	https://cran.r-project.org/package=segmented
ImageJ	NIH	https://imagej.net/ij/
ArcGIS 10.0	ESRI	http://www.esri.com/software/arcgis
SPSS	IBM	https://www.ibm.com/spss

Critical commercial assays		

GPS receiver	Garmin	eTrex Venture
Portable leaf-area meter	Yaxin (China)	Yaxin-1241
CHN elemental analyzer	PerkinElmer	2400 CHN
Industrial micro-focus X-ray micro-CT scanner	GE Sensing & Inspection Technologies	phoenix nanotom S
pH meter	WTW	SenTix® 81-87
H_2_SO_4_, HClO_4_, H_2_O_2_	Sigma-Aldrich	https://www.sigmaaldrich.cn/CN/zh/product/sigald/258105

**Other**		

PVC soil corer (50 mm inner diameter)	Custom	–
Soil sieves (1 mm, 0.25 mm)	Retsch	–

### Method details

#### Site description and sample collection

The study area lies within the Kalamaili Nature Reserve, China (88°30′ ∼ 90°03′ E, 44°36′ ∼ 46°00′ N, the total area is 14,856.48 km^2^), in the mid-latitudes of the Northern Hemisphere in the continental interior of Asia (Figure S1). The region has a temperate, continental arid climate, characterized by extreme dryness and scarce precipitation (mean annual rainfall 159.1 mm), frequent winds, and very high mean annual potential evapotranspiration (PET) of ∼2090.4 mm.^65^ As an ideal natural laboratory, its distinctive assemblage of gravel Gobi, low mountains, and sandy desert landforms^66^ creates a pronounced gradient in soil water content, providing favorable conditions for this study. The dominant soil type is calcareous Solonchaks.^67^ The desert flora exhibits community attributes typical of both Central Asian desert and the Mongolian Gobi, with distinctive morphology, anatomy, reproductive phenology, and interspecific interactions of considerable ecological value.^68^ Dominant species include *Haloxylon ammodendron*, *Krascheninnikovia ceratoides*, *Reaumuria songarica*, *Artemisia songarica*, *Anabasis salsa*, and *Stipa glareosa*.

Using a stratified random sampling approach, we distributed 13 sampling sites across gradients SWC. The sites did not differ in temperature or precipitation and were free from livestock grazing disturbance and other anthropogenic impacts. Sites were spaced at approximately 1 km intervals, and geographic coordinates and elevation were recorded with a handheld GPS (eTrex Venture; Garmin, Olathe, KS, USA). Field sampling was conducted from 15 to 20 August 2019. Along a SWC gradient spanning 38.8% (S1) to 8.1% (S13) at 13 sampling sites, within each sample site, five 10 m × 10 m quadrats were established, totaling 65 quadrats. For each plot, we recorded geographic coordinates (latitude–longitude), elevation, and slope. Both plant and soil samples were collected within the plots.

#### Vegetation survey, functional traits, and community indices

##### Vegetation survey

Within each plot, we identified all plant species and recorded individual counts. For each species, we measured two mutually perpendicular canopy diameters (D_1_, D_2_) and the maximum plant height (Hmax). Canopy projected area (A_canopy, m^2^) was approximated as an ellipse: A_canopy = π×(D_1_/2)×(D_2_/2). Species-level canopy coverage was then calculated as the sum of individual canopy areas divided by the plot area, expressed as a percentage (%). Total community cover was obtained by summing species-level covers, allowing for overlaps where applicable.

##### Plant functional traits and aboveground biomass measurement

We targeted three functional traits closely linked to plant life-history strategies: leaf area (LA, cm^2^), leaf nitrogen concentration (LN, mg g^-1^), and maximum plant height (Hmax, cm). For each plot, we randomly selected up to 10 mature individuals and collected 3∼5 fully expanded, non-senescent leaves per individual, with additional individuals sampled immediately outside the nearest plot when fewer than 10 of a given species were present, yielding 171 leaf samples in total. After field collection, leaves were gently rinsed with deionized water, blotted dry, and stored sealed in a cooler. LA was measured with a portable leaf-area meter (Yaxin-1241; Yaxin, China); when necessary, leaves were photographed against a scale and leaf area was computed in Image J. In the laboratory, all leaves were dried at 70 °C to constant mass, ground and passed through a 1-mm sieve (Retsch MM 400), and LN was determined with a PerkinElmer 2400 CHN elemental analyzer (PerkinElmer, USA). The AGB (g m^-2^) was determined by harvesting all aboveground tissues within each plot, and oven-drying samples at 65∼75 °C to constant mass (two consecutive weighings 12 h apart differing by <0.01 g; typically, 48∼72 h).

##### Plant community index calculations

**Species diversity**. The species richness index (S),^69^ the Shannon–Wiener index (H),^70^ and Simpson’s index (D)^71^ were used to characterize species richness and dominance in species’ relative abundances. We employed these three indices to quantify species diversity (hereafter SR). The Shannon–Wiener and Simpson indices were computed using (Equation 1), (Equation 2), respectively.

(Equation 1) H=−∑[(Pi)×ln(Pi)]

(Equation 2) $$D=1-\sum_{i=1}^{S}{P_{i}}^{2}$$

where *P*_*i*_ is the relative abundance of species *I*, and *S* is the total number of species. The above analysis was done using the “vegan” package in R software, version 4.0.3.^72^

**Functional diversity**. Community functional diversity (hereafter FD) was evaluated using plant leaf area (LA), maximum plant height (Hmax), and leaf nitrogen concentration (LN). We calculated three indices: functional dispersion (FDis), functional richness (FRic), and Rao’s quadratic entropy (RaoQ). FDis reflects the dispersion of functional traits among species; higher values indicate greater niche differentiation,^73^ and it was computed following Equation 3. FRic captures the extent to which species occupy the community’s functional (niche) space and the associated efficiency of resource use^74^; details are provided in Equation 4. RaoQ is used to assess diversity and variability within and among communities,^75^ with calculations given in (Equation 5), (Equation 6).

(Equation 3) $$FD\text{is}=\frac{\sum ajZj}{\sum aj}$$

where *aj* denotes the abundance of species *j*, and *Zj* is the distance from species *j* to the weighted centroid in trait space.

(Equation 4) $$FR\text{ic}=\frac{SFic}{Rc}$$

where *FRic* is the functional richness of trait *c* in community *i*. *SFic* is the ecological niche space of species in the community. *Rc* is the absolute trait value for different traits in different communities.

(Equation 5) $$R\text{aoQ}=\sum_{i=1}^{S-1}\sum_{j=i+1}^{S}d_{ij}P_{i}P_{i}$$

(Equation 6) $$d_{ij}=\frac{{u_{i}}_{j}}{n}$$

where *d*_*ij*_ is the distance between species *i* and species *j*; *P*_*i*_ is the proportion of species *i*; *n* is the total number of traits; *u*_*ij*_ is the number of species *i* and species *j* with different trait values. The above analysis was done using the “FD” package^50^ in R software, version 4.0.3.^72^

Community-weighted means. Community-weighted means (hereafter CWM) were obtained by averaging trait values (LA, LN, and Hmax) at the community level, weighted by species’ relative abundances.^18^ The parameter was calculated as:

(Equation 7) $$CWM=\sum Pi\times \text{tra}it_{i}$$

In the formula, where *Pi* is the relative abundance of species *i* in the community, *trait*_*i*_ is the trait value for species *i.* The above analysis was done using the “FD” package^76^ in R software, version 4.0.3.^72^

#### Soil sampling, X-ray microtomography, and soil properties

##### Soil sampling

After removing surface vegetation and litter from each plot, Intact rhizosphere soil cores were collected using PVC undisturbed corers (inner diameter 50 mm), one core per plot (total n = 65). During sampling, corers were advanced by gentle hand pressure and the surrounding soil was pared along the outer wall with a thin blade to minimize disturbance; Cores were double-sealed with Parafilm® and cling film, transported in insulated coolers, and stored at 4 °C until analysis.

##### Micro-CT scanning

We used a phoenix nanotom S industrial microfocus CT system (GE Sensing & Inspection Technologies, Wunstorf, Germany). Scan settings were: accelerating voltage 100 kV, tube current 100 μA, a 0.2-mm Cu filter to reduce beam hardening, and a rotation step of 0.3∼0.4°. Source–object distance (SOD) = 30 cm and source–detector distance (SDD) = 50 cm (object–detector distance = 20 cm), yielding a geometric magnification of M ≈ SDD/SOD ≈1.67. Reconstruction was performed in Datos|x 2.0 using a filtered back-projection (FBP) algorithm with ring-artifact and beam-hardening corrections enabled. Reconstructed slices were ∼2000 × 2000 pixels with isotropic voxels of 30 × 30 × 30 μm^3^. For downstream analyses, volumes were exported as 16-bit TIFF/DICOM and then isotropically downsampled by 2× to yield 60-μm voxels, after which a cubic 3-D ROI of 500 × 500 × 500 voxels (∼30 × 30 × 30 mm^3^) was cropped from the cylinder center to avoid wall effects.

##### Soil properties

SWC (%) was determined as follows^60^: after micro-CT scanning, plant roots, gravel, and debris were removed from each soil sample; the wet mass (ma) was measured to a precision of 0.01 g, the sample was oven-dried at 105 ± 2 °C to constant mass (typically ≥24 h), cooled in a desiccator, and the dry mass (ms) was recorded:

(Equation 8) $$SWC=\frac{\left(ma-ms\right)}{ms}\times 100$$

Total salinity (SSC, mg g^-1^): soil extracts were oven-dried at 105∼110 °C to constant mass (M1); organic matter was then removed from the dried material with H_2_O_2_ and the mass was recorded again (M2). SSC was calculated as the difference M1 − M2. Total nitrogen (TN, g kg^-1^): air-dried soils were finely ground and passed through a 0.25∼1.00 mm sieve, then analyzed by Kjeldahl digestion–distillation–titration using a Kjeltec System 2300 (FOSS/Tecator). Total phosphorus (TP, g kg^-1^): following H_2_SO_4_–HClO_4_ digestion, TP was determined spectrophotometrically using the molybdenum antimony–ascorbic acid reduction (molybdenum blue) method.^77^ pH: measured with a WTW glass electrode calibrated with pH 4.00, 7.00, and 10.00 buffers and equipped with temperature compensation.

##### Calculation of soil porosity and connectivity

Flat-field correction, 3D non-local means denoising, and histogram normalization were performed in ImageJ 1.53c. Pore–solid segmentation used Otsu’s global threshold (with Phansalkar’s local threshold applied for sensitivity checks), followed by a 3D morphological opening (spherical structuring element, radius = 1 voxel). A larger soil porosity (SP, %) indicates a greater pore volume^78^ and was computed according to Equation 9. Connectivity (PC, %) quantifies the structure of the pore network and reflects the spatial extent of pore influence within the soil. It was evaluated primarily from the relationship between the number of junctions and endpoints in the pore skeleton^79^ and calculated using Equation 10.

(Equation 9) $$\text{SP}=\frac{\sum V_{pores}}{\sum V_{ROI}}\times 100$$

(Equation 10) $$PC=\frac{J}{J+E}$$

where *V*_*pores*_ is the pore volume (mm^3^) and *V*_*ROI*_ is the volume of the entire soil-core ROI (mm^3^). *J* is the number of junctions, and *E* is the number of endpoints (excluding boundary endpoints).

### Quantification and statistical analysis

Exact sample sizes (n), definitions of n (e.g., number of plots/soil samples/individual plants), the statistical tests used, and measures of central tendency and dispersion (e.g., mean, median, SD, SEM, or confidence intervals) are reported in the corresponding figure legends and/or Results, with full test summaries provided in the Supplementary Tables (Tables S2, S3, S4, S5, S6, S7, S8, S9, S10, S11, S12, S13, S14, S15, S16, S17, and S18–S24). Unless otherwise stated, n denotes the number of plots (independent sampling units), and summary data are reported as mean ± SD; confidence intervals (when shown) are specified in the relevant figure legends.

#### Data screening and transformations

Prior to analysis, all data were subjected to Kolmogorov–Smirnov tests and Levene’s tests. Where necessary, variables were natural-log transformed to meet assumptions of normality and homoscedasticity.^80^ Prior to computing FD and CWM, species-level trait values were range-standardized to [0, 1] by variable, and Euclidean distances were used to reduce the influence of differing measurement scales. Outliers were identified using boxplots in conjunction with studentized residuals (|rstudent| > 3). Values deemed extreme but arising from plausible ecological conditions and compliant with measurement protocols were retained in modeling.

#### Site-level comparisons

Differences in soil properties and community attributes among sampling sites. Using site as a fixed factor, one-way ANOVAs were performed separately for soil variables and community metrics; when normality and homoscedasticity were satisfied, Tukey’s LSD was used for post-hoc comparisons. Lowercase letters in Figure 1 and Table 1 denote homogeneous groups from the multiple comparisons (shared letters indicate non-significant differences). These analyses were conducted in SPSS. Tables S2, S3, S4, S5, S6, S7, S8, S9, S10, S11, S12, S13, S14, S15, S16, and S17 report the descriptive statistics and one-way ANOVA summaries.

#### Effects of soil factors on plant community attributes

With soil variables as predictors and SR, FD, and CWM as responses, we performed detrended correspondence analysis (DCA) to evaluate maximum gradient lengths and select between redundancy analysis (RDA) and canonical correspondence analysis (CCA).^81^ Maximum gradient lengths were 1.05 (Soil–SR), 0.93 (Soil–FD), and 0.99 (Soil–CWM), all < 3, indicating suitability of RDA. Statistical inference used permutation tests with 9,999 permutations. Ordinations display only soil variables with significant effects (*p* < 0.05). These analyses were implemented in R using the package “vegan”.^82^ Tables S18–S20 report the significance tests of soil factors in the RDA.

#### Identifying drivers of AGB

Pairwise Pearson correlations were computed among all variables and visualized as a heatmap. To assess variable importance for AGB, we fitted bootstrap-aggregated random forest models (ntree = 500). Two permutation-based importance metrics were extracted: %IncMSE (percent increase in out-of-bag MSE upon permuting a variable), reflecting its contribution to predictive accuracy, and IncNodePurity (cumulative increase in node purity), reflecting fit improvements from splits. As random forests are nonparametric ensemble methods that do not yield parametric p-values for importance scores, linear associations and their significance were quantified via Pearson tests. To limit collinearity, variance inflation factors (VIFs) were computed; VIF > 10 indicated severe collinearity and 5 < VIF ≤ 10 moderate collinearity.^83^ Variables with VIF > 5 were excluded from subsequent SEMs, prioritizing ecologically interpretable and representative predictors; SWC, TN, and CWM-Hmax were retained as key factors. We tested the hypothesized causal structure in which SWC influences AGB both directly and indirectly via TN and CWM-Hmax (paths: SWC → TN, SWC → CWM-Hmax, TN → CWM-Hmax, and each of TN and CWM-Hmax → AGB; exogenous predictors were allowed to covary where appropriate). SEMs were fitted with lavaan using the MLR estimator (robust maximum likelihood with Satorra–Bentler corrections), FIML for missing data (missing = “fiml”), and standardized coefficients (std.all). Indirect effects and their uncertainty were estimated using nonparametric bootstrapping with 5,000 resamples and bias-corrected and accelerated (BCa) confidence intervals, implemented using the bootstrapLavaan () function in the R package semTools. Model adequacy was evaluated using the chi-square statistic (χ^2^), degrees of freedom (df), the comparative fit index (CFI), the Tucker–Lewis index (TLI), the root mean square error of approximation (RMSEA, reported with 90% confidence intervals), and the standardized root mean square residual (SRMR). We report standardized direct, indirect, and total effects; relative contributions were computed by normalizing absolute standardized total effects to sum to 100%. Path diagrams were produced with semPlot: semPaths (Tables S21–S23). All analyses in this subsection were conducted in R with “Hmisc”,^84^ “corrplot”,^85^ “randomForest”,^86^ “car”,^87^ “lavaan”,^88^ “semTools”,^89^ “semPlot”,^90^ “ggplot2”,^91^ “dplyr”,^92^ and “tidyr”.^93^

#### Driving mechanisms of the aridity gradient on AGB

Linear models and generalized additive models (GAMs) were used to evaluate AGB responses to SWC. Model selection favored the lower Akaike information criterion (AIC) value.^94^ Models were considered meaningfully different when ΔAIC > 2^95^ (Table S24). Upon confirming significant nonlinearity, segmented (piecewise) linear models were fitted for threshold diagnosis, indicating abrupt relationship changes beyond specific aridity levels. Threshold validity was assessed using likelihood-ratio tests (LRT) and the Davies test for changes in slope. To evaluate how the local marginal effects of SWC, CWM-Hmax, and their interaction on AGB vary along the SWC gradient, we specified a linear model (Equation 11). We then applied a moving-window analysis.^96^ Specifically, plots were ordered from low to high SWC. For each sliding window (15 adjacent plots), Equation 11 was re-fit using 100 bootstrap resamples, and the window-specific bootstrap coefficients were taken as estimates of local marginal effects. We set the SWC threshold at 28% (log10 = 1.447), overlaying separate linear trends on either side to depict piecewise behavior; in parallel, box-and-whisker panels summarized bootstrap coefficient distributions by window, with significant windows annotated by asterisks. Analyses were conducted in R using “mgcv”,^97^ “segmented”,^98^ “boot”,^99^ “dplyr”,^92^ “parallel”,^72^ “ggplot2”,^91^ and “chngpt”.^100^

(Equation 11) AGB ∼ CWM-Hmax + SWC + CWM-Hmax:SWC

#### Statistical significance and asterisk notation

Where statistical significance is indicated by asterisks in figures, the symbols denote the following thresholds unless otherwise stated: ∗ *p* < 0.05, ∗∗ *p* < 0.01, and ∗∗∗ *p* < 0.001. Asterisks correspond to the specific statistical tests described for each analysis, including one-way ANOVA with post hoc Tukey’s tests for site-level comparisons, permutation tests for RDA, Pearson correlation tests for bivariate associations, and parametric linear models, GAMs, segmented regression, or bootstrap-based inference for analyses along the SWC gradient.

## Supplemental information

Document S1. Figure S1 and Tables S1 and S18–S24

Table S2. One-way ANOVA for soil water content (SWC) among sampling sites

Table S3. One-way ANOVA for soil silt content (SSC) among sampling sites

Table S4. One-way ANOVA for total nitrogen (TN) among sampling sites

Table S5. One-way ANOVA for total phosphorus (TP) among sampling sites

Table S6. One-way ANOVA for pH among sampling sites

Table S7. One-way ANOVA for soil phosphorus (SP) among sampling sites

Table S8. One-way ANOVA for phosphorus content (PC) among sampling sites

Table S9. One-way ANOVA for species richness among sampling sites

Table S10. One-way ANOVA for Shannon-Wiener diversity index among sampling sites

Table S11. One-way ANOVA for Simpson diversity index among sampling sites

Table S12. One-way ANOVA for functional dispersion (FDis) among sampling sites

Table S13. One-way ANOVA for functional richness (FRic) among sampling sites

Table S14. One-way ANOVA for Rao’s quadratic entropy (RaoQ) among sampling sites

Table S15. One-way ANOVA for community-weighted mean of leaf area (CWM-LA) among sampling sites

Table S16. One-way ANOVA for community-weighted mean of maximum height (CWM-Hmax) among sampling sites

Table S17. One-way ANOVA for community-weighted mean of leaf nitrogen content (CWM-LN) among sampling sites
