# Supplementary material for: Divergent mechanisms governing aboveground biomass in desert plants across a drought gradient
Source: iScience. 2026 Mar 10;29(4):115304. doi: 10.1016/j.isci.2026.115304 (PMC13089035; doi:10.1016/j.isci.2026.115304)
Supplement: Document S1. Figure S1 and Tables S1 and S18–S24 [file mmc1.pdf]

iScience, Volume *29*

Supplemental information

Divergent mechanisms governing aboveground biomass in desert plants across a drought gradient

Kaiye Yuan, Hailiang Xu, Junjie Yan, and Guangpeng Zhang

Supplemental information

Supplementary Table 1. Important values of plant species in the field sites.

Important values	S1	S2	S3	S4	S5	S6	S7	S8	S9	S10	S11	S12	S13
Ephedra przewalskii	0.0	0.0	0.1	0.0	0.0	0.3	0.0	0.3	0.0	0.0	0.2	0.0	0.2
Anabasis salsa	0.0	0.0	0.0	0.0	0.0	0.0	0.0	0.0	0.0	0.1	0.0	0.3	0.1
Artemisia songarica	0.0	0.2	0.0	0.0	0.6	0.4	0.3	0.3	0.0	0.1	0.5	0.4	0.6
Stipa caucasica	0.0	0.1	0.0	0.0	0.0	0.0	0.1	0.1	0.0	0.7	0.1	0.0	0.0
Krascheninnikovia ceratoides	0.0	0.2	0.0	0.0	0.0	0.0	0.1	0.0	0.4	0.0	0.2	0.0	0.0
Haloxylon ammodendron	0.3	0.1	0.1	0.0	0.4	0.1	0.3	0.0	0.2	0.0	0.0	0.2	0.0
Reaumuria soongarica	0.0	0.0	0.0	0.0	0.0	0.0	0.3	0.0	0.0	0.1	0.0	0.1	0.0
Calligonum mongolicum	0.0	0.0	0.0	0.0	0.0	0.1	0.0	0.2	0.0	0.0	0.0	0.0	0.0
Kalidium foliatum	0.0	0.0	0.0	0.0	0.0	0.0	0.0	0.0	0.4	0.0	0.0	0.0	0.0
Tamarix chinensis	0.2	0.0	0.0	0.0	0.0	0.0	0.0	0.0	0.0	0.0	0.0	0.0	0.0
Halogeton glomeratus	0.0	0.0	0.0	0.0	0.0	0.0	0.0	0.0	0.0	0.0	0.0	0.0	0.0
Atraphaxis spinosa	0.2	0.0	0.0	0.0	0.0	0.0	0.0	0.0	0.0	0.0	0.0	0.0	0.0
Saussurea japonica	0.0	0.0	0.0	0.0	0.0	0.0	0.0	0.0	0.0	0.0	0.0	0.0	0.0
Suaeda physophora	0.0	0.0	0.0	0.0	0.0	0.0	0.0	0.0	0.0	0.0	0.0	0.0	0.0
Phragmites australis	0.2	0.0	0.0	0.0	0.0	0.0	0.0	0.0	0.0	0.0	0.0	0.0	0.0
Lepidium latifolium	0.1	0.0	0.0	0.0	0.0	0.0	0.0	0.0	0.0	0.0	0.0	0.0	0.0
Caragana microphylla	0.0	0.2	0.0	0.0	0.0	0.0	0.0	0.0	0.0	0.0	0.0	0.0	0.0
Achnatherum splendens	0.0	0.3	0.0	0.0	0.0	0.0	0.0	0.0	0.0	0.0	0.0	0.0	0.0
Artemisia terrae-albae	0.0	0.0	0.7	0.0	0.0	0.0	0.0	0.0	0.0	0.0	0.0	0.0	0.0
Allium polyrhizum	0.0	0.0	0.0	0.0	0.0	0.0	0.0	0.0	0.0	0.0	0.0	0.0	0.0
Salsola collina	0.0	0.0	0.0	1.0	0.0	0.0	0.0	0.0	0.0	0.0	0.0	0.0	0.0
Heteropappus altaicus	0.0	0.0	0.0	0.0	0.0	0.0	0.0	0.2	0.0	0.0	0.0	0.0	0.0
Zygophyllum fabago	0.0	0.0	0.0	0.0	0.0	0.0	0.0	0.0	0.0	0.0	0.0	0.0	0.0

Supplementary Table 18. Significance tests for soil factors in relation to community species diversity in the RDA. Df, Degrees of freedom; F, F statistic; *p*, P-value; p.signif, significance code for the adjusted P-value (ns, $p \geq 0.05$; •, $p \geq 0.1$;*, $p < 0.05$; **, $p < 0.01$; ***, $p < 0.001$).

Factors	Df	Variance explained	F	*p*	*p*.signif
SWC	1	1414.6	11.4	0.006	**
SSC	1	1016.1	8.2	0.020	*
TN	1	681.0	5.5	0.024	*
TP	1	57.6	0.5	0.506	ns
PH	1	800.0	6.4	0.016	*
SP	1	501.4	4.0	0.050	*
PC	1	136.2	1.1	0.268	ns

Supplementary Table 19. Significance tests for soil factors in relation to community functional diversity in the RDA.

Factors	Df	Variance explained	F	*p*	*p*.signif
SWC	1	1.3	33.1	0.002	**
SSC	1	0.2	5.2	0.012	*
TN	1	0.0	0.4	0.594	ns
TP	1	0.0	0.3	0.712	ns
PH	1	0.1	3.4	0.056	•
SP	1	0.2	4.1	0.028	*
PC	1	0.0	0.2	0.788	ns

Supplementary Table 20. Significance tests for soil factors in relation to community-weighted means in the RDA.

Factors	Df	Variance explained	F	*p*	*p*.signif
SWC	1	238.2	55.2	0.002	**
SSC	1	14.2	3.3	0.084	•
TN	1	22.1	5.1	0.028	*
TP	1	1.5	0.3	0.552	ns
PH	1	5.5	1.3	0.256	ns
SP	1	3.2	0.7	0.382	ns
PC	1	3.6	0.8	0.340	ns

Supplementary Table 21. Structural equation model linking SWC, TN, CWM-Hmax and AGB. Unstandardized estimates (Est), path label (a, b, c, …), standard errors (SE), z statistics, *p* values, and standardized estimates (Std.Est) are shown. conf.low and conf.high, lower and upper bounds of the 95% confidence interval by 1000 bootstrap resamples.

Response	Predictor	Label	Est	SE	z	*p*	Std.Est	conf.low	conf.high
TN	SWC	a	0.454	0.111	4.105	<0.001	0.454	0.237	0.670
CWM-Hmax	TN	b	0.224	0.100	2.239	0.025	0.224	0.028	0.420
CWM-Hmax	SWC	c	0.565	0.100	5.650	<0.001	0.565	0.369	0.761
log(AGB)	CWM-Hmax	d	1.162	0.310	3.750	<0.001	0.461	0.554	1.769
log(AGB)	SWC	e	0.759	0.305	2.491	0.013	0.302	0.162	1.357
log(AGB)	TN	f	0.011	0.259	0.041	0.968	0.004	−0.497	0.518

Supplementary Table 22. Indirect and total effects from the structural equation model. Effects were defined in the lavaan syntax (labels). Unstandardized estimates (Est), standard errors (SE), z statistics, *p* values, standardized estimates (Std.Est), and 95% confidence intervals (CI) are shown.

Effect label	Description	Est	SE	z	*p*	Std.Est	conf.low	conf.high
SWC_TN_CWM-Hmax	SWC → TN → CWM-Hmax	0.102	0.052	1.966	0.049	0.102	0	0.203
SWC_CWM-Hmax_AGB	SWC → Hmax → AGB	0.656	0.21	3.124	0.002	0.261	0.245	1.068
SWC_TN_CWM-Hmax_AGB	SWC → TN → CWM-Hmax → AGB	0.118	0.068	1.741	0.082	0.047	−0.015	0.251
TN_CWM-Hmax_AGB	TN → CWM-Hmax → AGB	0.26	0.135	1.922	0.055	0.103	−0.005	0.525
SWC_TN_AGB	SWC → TN → AGB	0.005	0.118	0.041	0.968	0.002	−0.226	0.235
SWC_AGB_indirect	Total indirect effect of SWC on AGB	0.779	0.235	3.32	0.001	0.309	0.319	1.239
SWC_AGB_total	Total effect of SWC on AGB	1.538	0.247	6.223	<0.001	0.611	1.054	2.023

Supplementary Table 23. Model fit indices and coefficient of determination (R^2) for the structural equation model. Effects were defined in the lavaan syntax (labels). Unstandardized estimates (Est), standard errors (SE), z statistics, *p* values, standardized estimates (Std.Est), and 95% confidence intervals (CI) are shown.

**Index**	**Value**
χ^2	0
df	0
CFI	1
RMSEA	0
SRMR	0
AIC	586.352
**Variable**	**R^2**
TN	0.206
CWM-Hmax	0.484
log(AGB)	0.492

Supplementary Table 24. Best model for the relationship between AGB and SWC. Linear is Linear models. GAM is Generalized additive model. Lower AIC values indicate a better fit of the model. R^2, Correlation index, the larger the value, the better the regression effect.

**Variable**	**AIC**		**R^2**	
	Linear	GAM	Linear	GAM
AGB and SWC	54.24	-2681.90	0.07	1

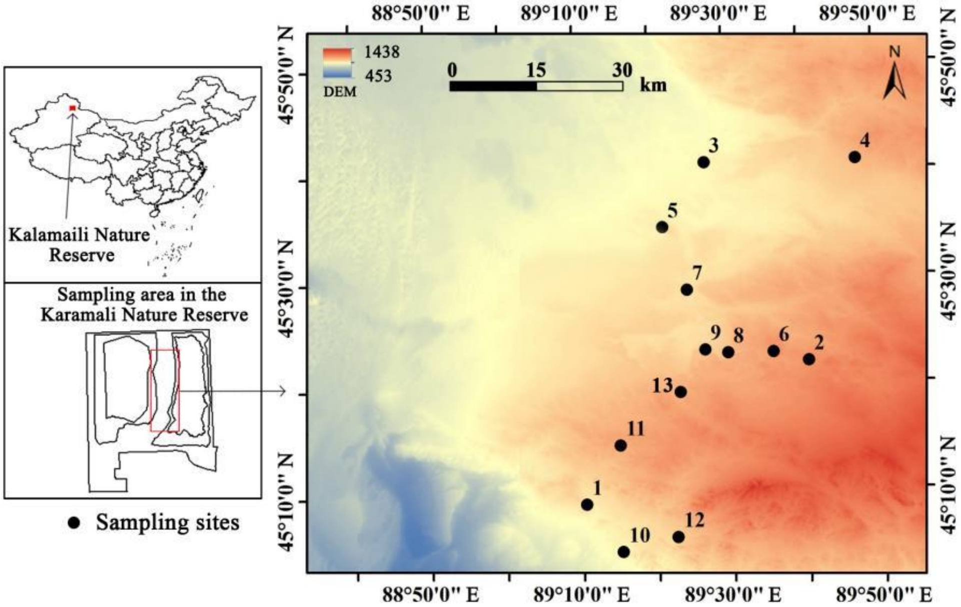

Supplementary Figure 1. Sampling sites. This figure was originally generated using the ArcGIS 10.0 software (Environmental Systems Research Institute, CA, USA. http://www.esri.com/software/arcgis/arcgisonline (accessed on 1 January 2022)).
